# The Toxicity of Normal Urine

**Published:** 1885-09

**Authors:** 


THE TOXICITY OF NORMAL URINE.

The toxicity of normal urine has been a disputed question for a long period of time. The weight of evidence is on the affirmative side, although the immediate poisonous principles are not known. Urea, uric acid, kreatin, and the potassium salts have in turn been incriminated. Recent investigations, however, point to the existence of poisonous alkaloids in normal urine. M. CHAUTEMESSE has summarized the history of the subject, which is well abstracted in two leading articles, appearing in the *British Medical Jorunal*, June 6th and July 4th, 1885.

Alkaloids have been found in putrid albumens, bile and normal muscle-juice by GAUTIER and POUCHET. BROUARDEL and BOTUMY published, in 1881, a distinguishing reaction between the vegetable alkaloids and the ptomaines. The alkaloids of putrefacton, in the presence of potassium ferricyanide and ferric chloride yield the Prussian blue coloration. In 1880, POUCHET found an alkaloid substance in normal urine, which, in combination with hydrochloric acid, formed double salts with platinum, gold and mercury. BROUARDEL'S discovery that "alkaloids exist in the bodies of living beings, which have been generated in the alimentary canal, and probably elaborated by the vegetable organisms there present acting as the agents in intestinal putrefactions," marks an important advance beyond the results of the labors of SELMI, GAUTIER, POUCHET and BOUTMY. Part of these intestinal alkaloids are absorbed into the blood, further elaborated by the kidneys, and appear in the urine.

Professor BOUCHARD, at a recent meeting of the Sociétè de Biologie, read an interesting and valuable paper on the toxic qualities of normal urine. In the light of the recent progress of physiological chemistry, briefly detailed in this paper, the results of Professor BOUCHARD'S experiments are clearly intelligible. The fact of toxicity of the urine was established by the injection of specimens of urine into the veins of frogs and rabbits.

Following the injection of normal urine into the veins of a rabbit, BOUCHARD observed "contraction of pupils, slow respiration, muscular weakness, lowered temperature, abolition of reflexes, torpor," and death as the result of arrest of respiration. It is a well-known fact that 90 cubic centimètres of water for every kilogramme of its body-weight may be injected into the veins of an animal without the production of mechanical effects. In BOUCHARD'S experiments, the quantity of fluid used was not the tenth of this amount, so that the effects were of a true toxic character.

BOUCHARD next attempted to determine the particular constituent to which the poisonous character was due. Without entering into the details of the experiment, it was found that urea, uric acid, kreatin, and the potassium salts, alone and combined, "though undoubtedly poisonous, were not so in the small quantities contained in the urine injected. He concludes that there are numerous poisonous principles present, which do not reside in one, but in several of the urinary constituents." The poisonous principles are not volatile.

Very recently, LEPINE and GUERIN have demonstrated that these alkaloid bodies are increased in various acute diseases. Thus in typhoid fever and pneumonia, the toxicity of the urine was greatly increased, while in the urine of diabetes, catarrhal jaundice, and cirrhosis with jaundice no increase of toxicity was observed.—*Journal of the American Medical Association.*

